# Phylogenetic Footprints of Coagulase‐Negative Staphylococci and *Mammaliicoccus* Isolated From Raw Caprine Milk

**DOI:** 10.1002/mbo3.70171

**Published:** 2025-12-23

**Authors:** Ahmed B. Omer, Kamal H. Eltom, Adam Bashir Tawor, Osman Erganiş

**Affiliations:** ^1^ Department of Microbiology Al‐Rass Food Safety Laboratory, Alqassim Saudi Arabia; ^2^ Department of Animal Health and Safety of Animal Products, Institute for Studies and Promotion of Animal Exports University of Khartoum, Shambat Khartoum North Sudan; ^3^ Department of Microbiology, Faculty of Veterinary Medicine University of Selçuk Konya Türkiye; ^4^ Department of Microbiology, Faculty of Veterinary Science University of Al Gadarif Al‐Gadarif Sudan

**Keywords:** coagulase‐negative, microbial genomics, One Health, phylogenetic analysis, *Staphylococcus*, *tuf* gene

## Abstract

Coagulase‐negative staphylococci (CNS) and *Mammaliicoccus* species recently reclassified from the *Staphylococcus sciuri* group, are increasingly recognized as opportunistic pathogens in dairy animals and humans. This study investigated phylogenetic diversity and antimicrobial resistance in raw caprine milk from Sudan by integrating conventional bacteriological methods, molecular sequencing, and antimicrobial susceptibility testing. Raw goat milk samples were cultured, and presumptive CNS isolates were identified using phenotypic tests (novobiocin, oxidase, urease, and carbohydrate fermentation) following the standard *Staphylococcus* identification flow chart. PCR amplification of the elongation factor Tu (*tuf*) gene and the methicillin‐resistance gene (*mecA*) enabled molecular confirmation and assessment of antimicrobial resistance. Sequenced *tuf* amplicons (~370 bp) were analyzed by BLAST and aligned in MEGA 12 for maximum‐likelihood phylogenetic reconstruction. *Staphylococcus simulans* and *Mammaliicoccus lentus* were isolated in this study; antimicrobial susceptibility testing revealed that *S. simulans*, but not *M. lentus*, was methicillin‐resistant and carried the *mecA* gene. Partial *tuf* gene sequencing confirmed 99.6%–99.7% identity with reference strains of the respective species. Phylogenetic analysis revealed that the isolated *S. simulans* formed a distinct branch within the global clusters. Meanwhile, *M. lentus* was found to be closely related to the global strains, showing only minor divergence. This study reports the presence of *S. simulans* and *M. lentus* in caprine milk from Sudan using both phenotypic and genotypic identification methods. This underscores the importance of integrating traditional laboratory methods with molecular techniques for precise species identification. The identification of methicillin‐resistant *S. simulans* highlights the need for ongoing monitoring of CNS in raw milk.

## Graphical abstract

Coagulase‐negative Staphylococci and *Mammaliicoccus* were isolated from raw caprine milk and confirmed molecularly, while their antimicrobial resistance profiles were determined using the disk diffusion method. Notably, *M. lentus* and *S. simulans* exhibited genetic divergence from global strains, emphasizing the relevance of a One Health approach in mastitis surveillance and antimicrobial resistance monitoring.

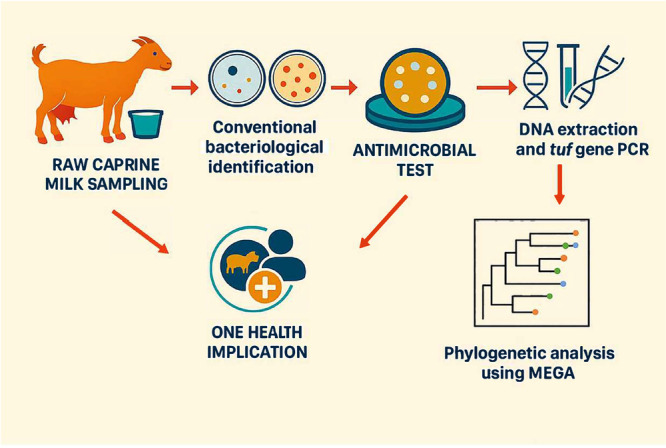

## 1 Introduction

In recent taxonomic revisions, five species formerly classified within the *Staphylococcus sciuri* group, *Staphylococcus sciuri*, *S. fleurettii*, *S. lentus*, *S. stepanovicii*, and *S. vitulinus*, have been reassigned to a newly established genus, *Mammaliicoccus*. *Mammaliicoccus sciuri* has been designated as the type species of this genus, marking a significant shift in the classification of these coagulase‐negative staphylococci (CNS) (Madhaiyan et al. 2020). Staphylococci and mammaliicocci are considered opportunistic pathogens that can cause severe infections (Belhout et al. 2022; Nemeghaire et al. 2014).

CNS represent a heterogeneous group of Gram‐positive bacteria that are ubiquitous and commonly found as commensals on the skin and mucous membranes of humans and animals (Piette and Verschraegen 2009). Within the genus *Staphylococcus*, these species are distinguished from coagulase‐positive staphylococci, such as *Staphylococcus aureus*, based on their inability to produce the coagulase enzyme, a key virulence factor traditionally used in diagnostic microbiology (Ogodo 2022; Gebremedhin et al. 2022). Historically, CNS were considered non‐pathogenic contaminants due to their frequent isolation from healthy skin and environmental sources. However, accumulating evidence has redefined their role in both human and veterinary medicine, demonstrating their capacity to act as opportunistic pathogens under favorable conditions.

In veterinary contexts, particularly in dairy production systems, CNS have emerged as important etiological agents of intramammary infections (IMI) (Getahun et al. 2024). While *S. aureus* remains the primary cause of clinical mastitis in cattle, CNS is more commonly associated with subclinical mastitis in small ruminants such as goats. Subclinical mastitis, although often asymptomatic, can significantly impair milk yield and quality, elevate somatic cell counts, and affect animal welfare and farm profitability. Goats are especially susceptible due to their unique udder anatomy and milking practices, which may facilitate the entry and colonization of these bacteria (Gelasakis et al. 2016).

Numerous CNS species have been isolated from caprine milk, with species such as *S. chromogenes*, *S. epidermidis*, *S. caprae*, *S. haemolyticus*, *S. simulans*, and *S. xylosus* frequently reported in both healthy and infected mammary glands (Clark and Mora García 2017; Koop et al. 2010). Although these species generally exhibit lower virulence compared to *S. aureus*, they possess several adaptive mechanisms, including biofilm formation, antibiotic resistance, and host immune evasion, that allow them to persist in the mammary gland environment. The ability of the CNS to form biofilms is particularly concerning, as it contributes to chronic infection, treatment failure, and the potential for transmission through raw milk or dairy products (Goetz et al. 2017).

Raw caprine milk provides a unique ecological niche for CNS, functioning both as a reservoir and a vehicle for bacterial dissemination (Koop et al. 2012). The presence of CNS in milk not only reflects udder health but may also pose risks to public health, particularly when raw milk is consumed or processed without adequate thermal treatment (Bernier Gosselin et al. 2019). Moreover, increasing reports of antimicrobial‐resistant CNS strains in dairy herds have raised concerns about the potential role of animal‐associated CNS as reservoirs of resistance genes, which could be transferred to more virulent staphylococcal or Mammaliicoccal species through horizontal gene exchange (Virdis et al. 2010; Nelli et al. 2022).

Despite the growing awareness of CNS in goat mastitis, relatively few studies have explored their phylogenetic diversity or evolutionary dynamics in the context of raw caprine milk. This gap is particularly evident in regions such as Sudan, where goat milk is an important nutritional and economic resource; however, it has not been extensively studied from microbiological or genomic perspectives (Mohamedsalih and Abubaker 2021). Moreover, the zoonotic potential of CNS and *Mammaliicoccus* species isolated from raw milk remains poorly understood, particularly in communities where unpasteurized dairy products are commonly consumed. This represents a significant One Health concern, as antimicrobial‐resistant strains may circulate between animals and humans, especially in rural settings or among immunocompromised individuals (Claeys et al. 2013; Suresh 2020).

Understanding their genetic diversity and evolutionary trajectories is critical for unraveling their pathogenic potential and developing targeted diagnostic and control strategies. Phylogenetic analysis offers a powerful framework for elucidating the evolutionary relationships among bacterial strains. By comparing genetic sequences across taxa, this approach enables the reconstruction of lineage structures, the identification of host‐specific adaptations, and the tracing of gene flow, including antimicrobial resistance and virulence determinants (Pickering et al. 2021). In the context of mastitis pathogens, phylogenetic tools can reveal how particular CNS lineages adapt to the caprine host and persist within the mammary gland environment, thereby informing epidemiological surveillance and intervention strategies (Schauer et al. 2021).

In this study, we used partial *tuf* gene sequencing, phylogenetic tree reconstruction, and antimicrobial susceptibility testing to characterize the isolates. This integrative approach provides both evolutionary and clinical insight into CNS and *Mammaliicoccus* strains circulating in raw goat milk (Becker et al. 2014; Carter 2018).

This study aims to investigate the phylogenetic footprints of CNS and *Mammaliicocci* isolated from raw caprine milk. By applying molecular and phenotypic characterization techniques, we seek to elucidate the diversity, genetic relatedness, and antimicrobial resistance profiles of isolates. These findings will improve our understanding of the epidemiology and evolutionary dynamics of CNS species in goat populations. The findings will also provide insights to improve mastitis diagnosis and treatment decisions, as well as enhance milk safety and quality for consumers.

## 2 Materials and Methods

**Figure 1:**
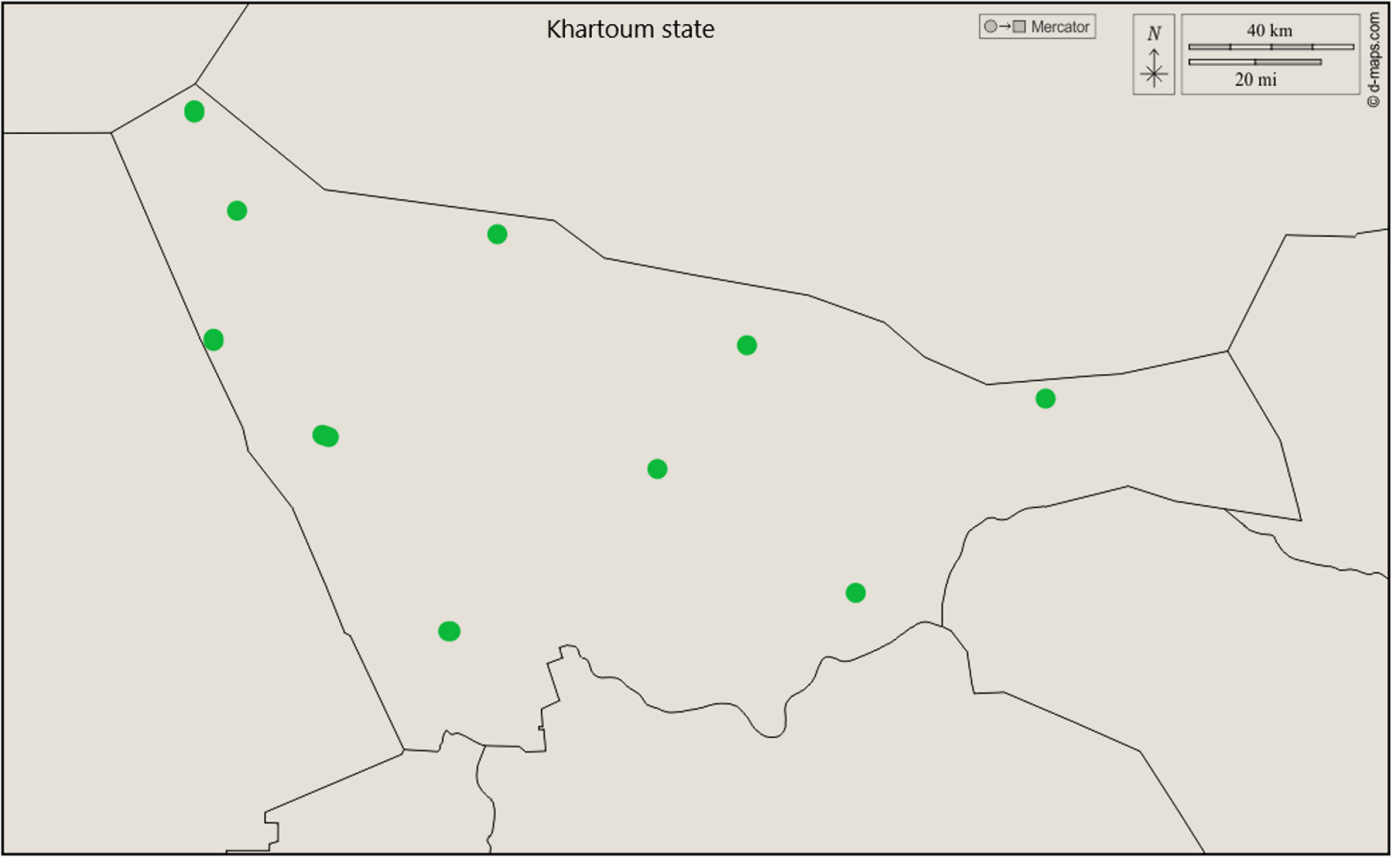
Sampling areas in Khartoum state—Sudan.

A total of 200 raw milk samples from goats were collected for the isolation of bacterial pathogens in Khartoum, Sudan (Figure 1). Samples were cultured on blood agar, and pure isolates were confirmed on nutrient agar. Then, coagulase‐negative isolates were detected by conventional methods as described in the Flow Chart for the Identification of *Staphylococcus* spp. by Elsanousi (El Sanousi 2015), which included oxidase, urease, and carbohydrate fermentation tests for mannose, sucrose, trehalose, ribose, xylose, and raffinose. Media were prepared according to standard protocols and incubated at 37°C. The tests facilitated preliminary identification of staphylococcal species involved in caprine mastitis.

### 2.1 Molecular Identification

#### 2.1.1 DNA Extraction

DNA extraction from *Staphylococcus* and *Mammaliicoccus* isolates was carried out using QIAamp DNA Mini Kit from Qiagen (Hildesheim, Germany) according to the instructions on the product sheet.

#### 2.1.2 PCR Master Mixture

The PCR was performed in a 20 μL reaction volume containing 3 μL of DNA template, 10 pM of each primer, 1 U of *iTaq* DNA polymerase (Intron, Seoul, Korea), 2 μL of dNTPs mixture (2.5 mM each), and 2 μL of 10 buffer (containing 100 mM Tris HCl, pH 8.3, 500 mM KCl, 20 mM MgCl_2_).

#### 2.1.3 Polymerase Chain Reaction (PCR) Conditions

The DNA amplification was carried out as follows: an initial denaturation step at 94°C for 3 min, followed by 30 cycles of denaturation at 94°C for 30 s, annealing at 55°C for 30 s, extension at 72°C for 30 s, and a final step of extension at 72°C for 5 min. The PCR products were analyzed by electrophoresis in agarose gel after staining.

#### 2.1.4 Primers

Amplification of the *tuf* gene was performed using the primer pair: TStaG422 (5′‐GGCCGTGTTGAACGTGGTCAAATCA‐3′) and TStaG765 (5′‐TIACCATTTCAGTACCTTCTGGTAA‐3′). These are specific to conserved regions of the *tuf* gene commonly found in staphylococci and closely related genera such as *Mammaliicoccus*, as described (Madhaiyan et al. 2020; Martineau et al. 1998).

For the detection of the *mecA* gene, the primers *mecA*1 (5′‐AAAATCGATGGTAAAGGTTGGC‐3′) and *mecA*2 (5′‐AGTTCTGCAGTACCGGATTTGC‐3′) were used according to the protocol by (Strommenger et al. 2006).

#### 2.1.5 Agarose Gel Electrophoresis

PCR amplification results of different primers were analyzed by subjecting 8 μL of the PCR product to electrophoresis in 1% agarose gel stained with RedSafe stain (Intron, Seoul, Korea) and visualized by UV trans‐illuminator. The visualized products were recorded and captured in a gel documentation system (Major Science, Seoul, Korea).

### 2.2 Antibiotic Susceptibility Test

Antimicrobial susceptibility testing was performed using the disk diffusion method. Fresh colonies from nutrient agar were inoculated into nutrient broth and incubated at 37°C for 18 h. The bacterial suspension was then adjusted to approximately 0.5 McFarland turbidity standard and uniformly streaked onto Mueller–Hinton agar (HiMedia, Mumbai, India) using a sterile cotton swab. Antibiotic discs containing 5 µg of methicillin or novobiocin (Bioanalyse Ltd., Ankara, Turkey) were placed on the surface, and plates were incubated at 37°C for 24 h. After incubation, inhibition zone diameters were measured in millimetres and interpreted as susceptible, intermediate, or resistant according to the manufacturer's instructions and Clinical and Laboratory Standards Institute (CLSI) guidelines.

### 2.3 Sequencing

The *tuf* gene primer amplification products were sent to a commercial company (Macrogen, Seoul, Korea) for sequencing using the amplification primers. Obtained sequences were edited using the BioEdit sequence alignment editor. The obtained sequences were aligned using the Basic Local Alignment Search Tool (BLAST) from the National Center for Biotechnology Information (NCBI) in the United States. This was done in conjunction with the available CNS *tuf* gene sequences that were deposited in GenBank.

### 2.4 Data Analysis and Phylogenetic Tree Construction: Molecular Phylogenetic

The resulting sequences for CNS identified in this study were used to construct phylogenetic trees using MEGA version 12 (Kumar et al. 2024). Sequences of the respective type strains, as well as sequences of representative related species worldwide obtained from GenBank and NCBI, were included in each tree.

### 2.5 Evolutionary Analysis by the Maximum Likelihood Method

The phylogeny was inferred using the Maximum Likelihood method and the model (Tamura and Nei 1993). The tree displayed, which has the highest log likelihood (−1706.31), was constructed based on nucleotide substitutions. The initial tree for the heuristic search was chosen by selecting the one with the better log‐likelihood between a neighbor‐joining (NJ) tree (Saitou and Nei 1987) and a maximum parsimony (MP) tree. The NJ tree was generated using a matrix of pairwise distances computed using the p‐distance (Nei and Kumar 2000). The MP tree with the shortest length was selected from 10 MP tree searches, each initiated from a randomly generated starting tree. The analysis included seven nucleotide sequences comprising 1191 positions in the final data set. Evolutionary analyses were performed using MEGA12 (Kumar et al. 2024) utilizing up to three parallel computing threads.

## 3 Results

### 3.1 Conventional Bacteriology Results

Following the Flow Chart for the Identification of *Staphylococcus* species (El Sanousi, 2015), 15 *Staphylococcus* spp. were isolated (7.5%) among them five isolates were coagulase negative, two isolates were *S*. *simulans* isolates and three isolates were *M. lentus*. Although *M. lentus* has been reclassified into a different genus, it still shows the same phenotypic characteristics. *Staphylococcus* and *Mammaliicoccus* species were identified and then characterized; *M. lentus* showed novobiocin resistance, negative reactions for both oxidase and urease, and the ability to ferment raffinose. In contrast, *S. simulans* was novobiocin‐sensitive and oxidase‐negative. These distinct biochemical profiles aid in the differentiation and confirmation of species identity.

### 3.2 Susceptibility to Methicillin

Phenotypic antimicrobial susceptibility testing was performed using the disc diffusion method, as outlined in the CLSI guidelines. All three isolates of *M. lentus* were found to be phenotypically susceptible to methicillin, while *S. simulans* isolates exhibited resistance. This phenotypic resistance in *S. simulans* prompted further molecular investigation to confirm the presence of methicillin resistance genes.

### 3.3 Molecular Biology Results

#### 3.3.1 Detection of the *tuf* Gene by PCR

**Figure 2:**
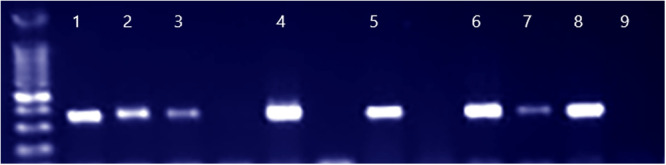
PCR amplification of the *tuf* gene from *M. lentus* and *S. simulans* isolates on 1% agarose gel. Electrophoresis was performed on a 1% agarose gel, and DNA bands were visualized under UV illumination after DNA staining. Lane 1: positive control; Lanes 2–8: *M. lentus* and *S. simulans* isolates; Lane 9: negative control. The expected amplicon size was 370 bp.

All 15 isolates were subjected to PCR amplification targeting the *tuf* gene, which encodes elongation factor Tu, a highly conserved protein across staphylococci. The *tuf* gene is widely used as a genus‐specific marker for the molecular identification of staphylococcal species, as well as genetically related species, such as those in the *Mammaliicoccus* genus, which was recently reclassified from the *Staphylococcus sciuri* group. PCR analysis showed 100% successful amplification in all tested isolates, indicating their taxonomic affiliation within the genus *Staphylococcus* or *Mammaliicoccus* (Figure 2).

#### 3.3.2 Detection of the Methicillin Resistance Gene (mecA)

**Figure 3:**
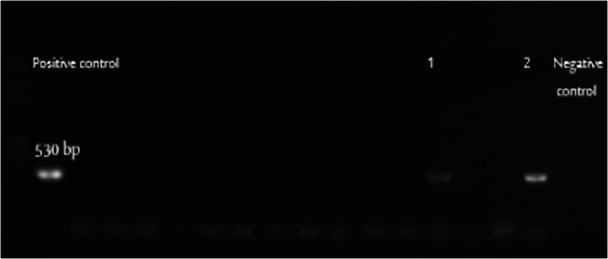
PCR amplification of the *mecA* gene from *Staphylococcus simulans* and *M. lentus* isolated from caprine milk. Amplification products were separated by electrophoresis on a 1% agarose gel and visualized under UV light. The expected amplicon size was 530 bp. Two *S. simulans* isolates showed positive amplification for the *mecA* gene, confirming methicillin resistance.

**Figure 4:**
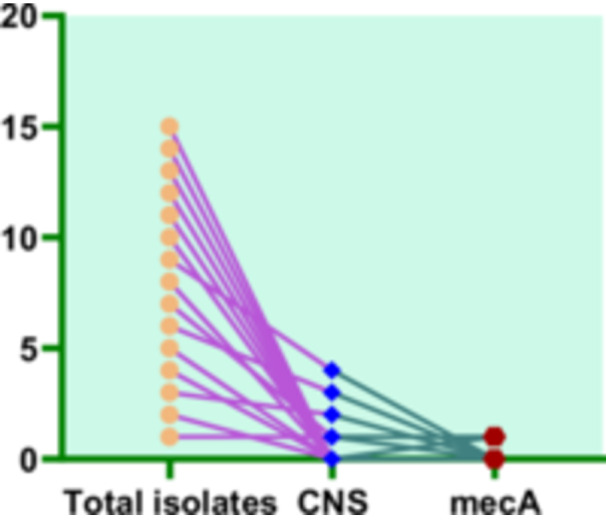
Distribution of CNS among *Staphylococcus* isolates. Graph explaining the distribution of CNS isolates after molecular confirmation using *tuf* gene as housekeeping gene, also showing the *mecA gene* existence in isolated CNS.

To determine the genetic basis of methicillin resistance, all the five CNS isolates were screened for the mecA gene, which encodes an alternative penicillin‐binding protein (PBP2a) associated with resistance to β‐lactam antibiotics. PCR amplification revealed that only the two isolates of *S. simulans* were positive for mecA, confirming their methicillin‐resistant status as observed in phenotypic testing (Figures 3 and 4).

### 3.4 Partial Sequencing and Analysis of the *tuf* Gene

Tow samples (*S. simulans* and *M. lentus*) were sent for sequencing a partial segment (~370 base pairs) of the *tuf* gene was amplified using forward and reverse primers. The PCR products were purified, sequenced in both directions, and aligned using sequence editing software. The high‐quality consensus sequences were then subjected to a BLASTn search (NCBI) for species identification based on sequence similarity. The analysis showed 100% identity with reference sequences for *Mammaliicoccus lentus* (formerly *Staphylococcus lentus*) and *Staphylococcus simulans*.

### 3.5 Phylogenetic Relationship Between CNS Isolates and Reference Strains

#### 3.5.1 Staphylococcus simulans

**Figure 5:**
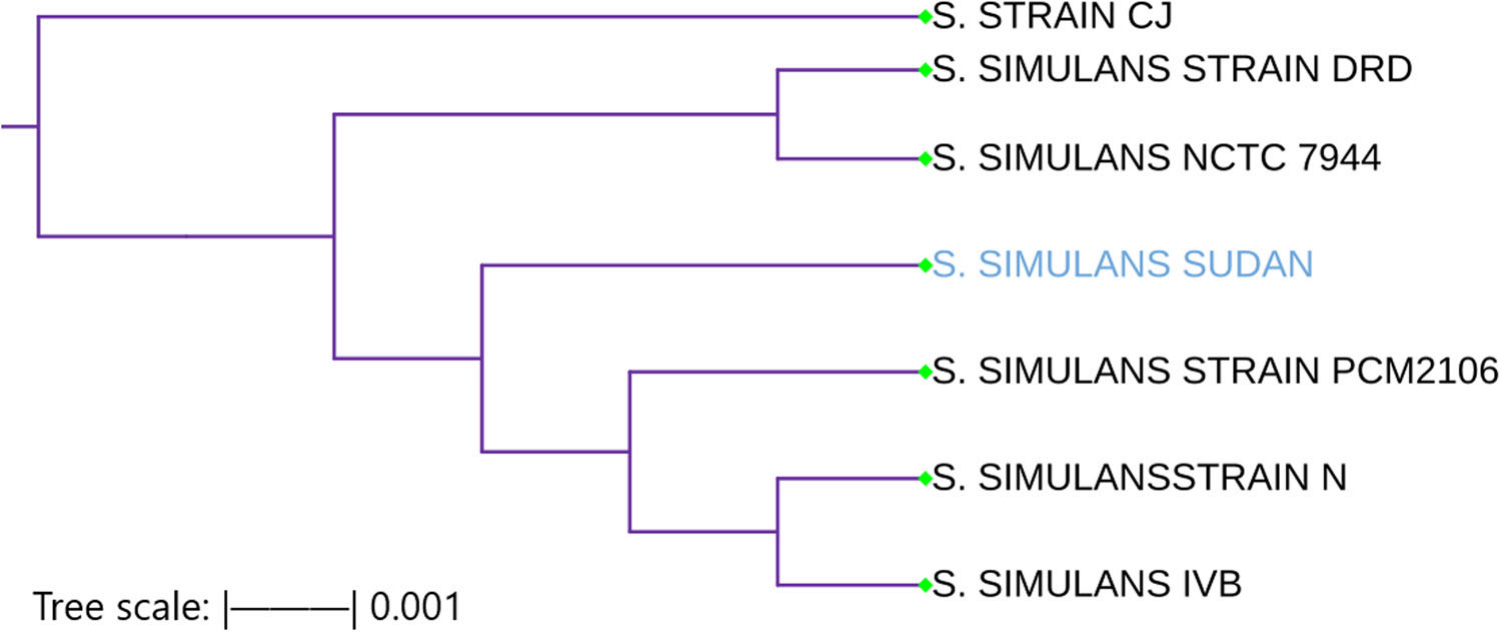
Phylogenetic relationship of the Sudanese *Staphylococcus simulans* isolate based on *tuf* gene sequences. Maximum Likelihood phylogenetic tree of partial *tuf* gene sequences (1,191 bp) inferred using MEGA12 (best log likelihood = –1706.31). The Sudanese *S. simulans* isolated from subclinical mastitis (blue) clusters within a clade of *S. simulans* reference strains from GenBank.

The *tuf* gene sequence of *Staphylococcus simulans* identified in this study showed 99.6% sequence identity with reference strains *S. simulans* strain PCM 2106 (accession no. MF679022.1) and *S. simulans* strain CJ16 (accession no. KP325407.1) isolated from Zhejiang, China. In the phylogenetic analysis, this isolate clustered closely with its most genetically related strains worldwide, confirming its species‐level identification. The phylogenetic tree demonstrates strong bootstrap support for this clustering, as illustrated in Figure 5.

#### 3.5.2 *Mammaliicoccus lentus* (formerly classified as *Staphylococcus lentus*)

**Figure 6:**
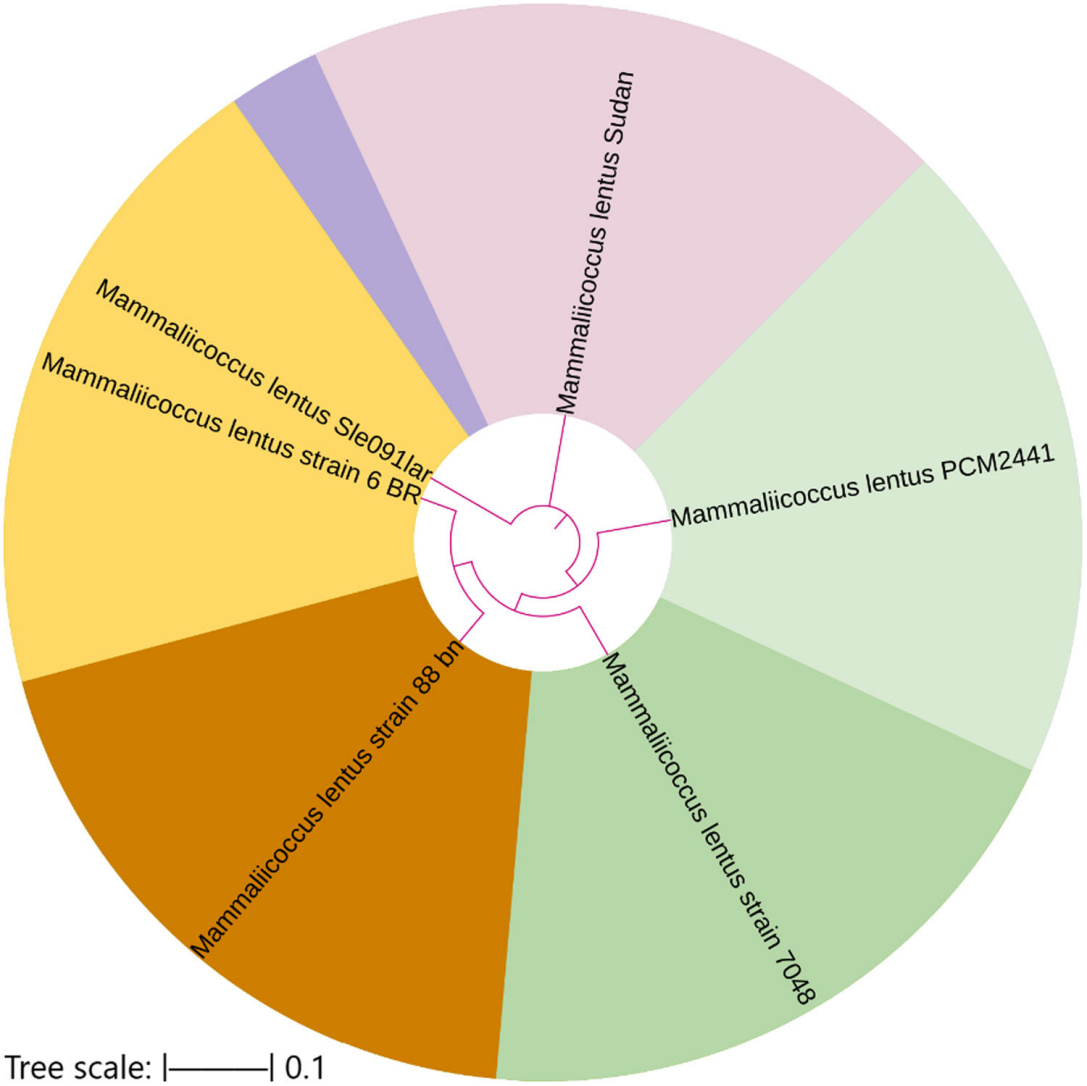
Phylogenetic position of the Sudanese *M. lentus* isolate. The Sudanese *M. lentus* isolate forms a distinct branch, showing clear divergence from other strains and suggesting unique regional or genomic characteristics. This highlights the species phylogenetic diversity and the potential epidemiological significance of the Sudanese.

The sequence of the *M. lentus* isolate identified in this study exhibited 99.68% identity with *M. lentus* strain Sle‐091lar (previously named *S. lentus*, accession no. MG557993.1), which was isolated from Larissa, Greece. This isolate also clustered phylogenetically with closely related global strains, supporting the taxonomic assignment to the genus *Mammaliicoccus*. Its position within the tree is supported by high sequence similarity and phylogenetic confidence, as shown in Figure 6.

#### 3.5.3 *M. lentus* and *S. simulans*

**Figure 7:**
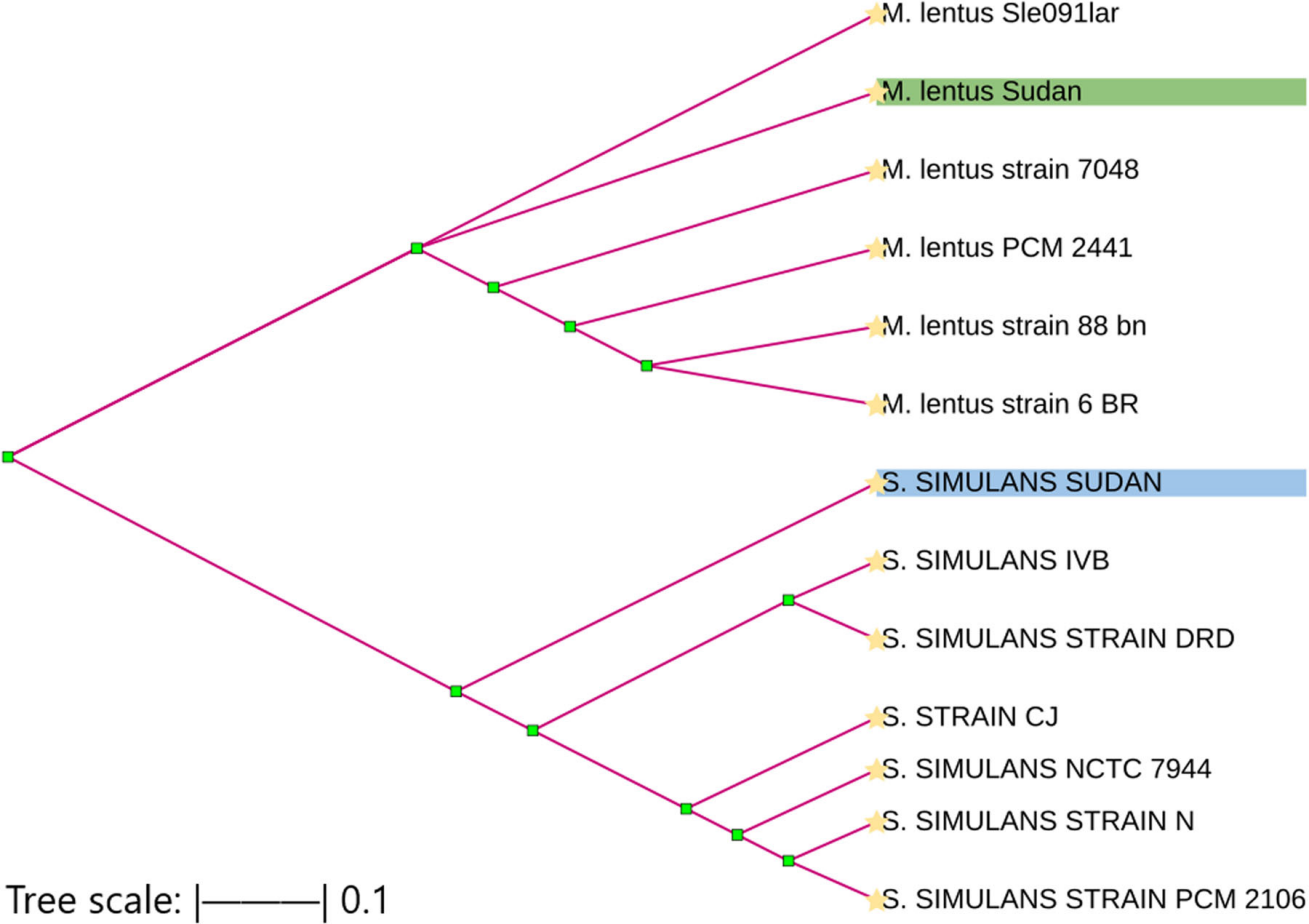
The *tuf* gene‐based phylogenetic tree for *M. lentus* and *S. simulans.* The *tuf* gene‐based phylogenetic tree shows two distinct clades: *Mammaliicoccus lentus* and *Staphylococcus simulans*. Sudanese isolates cluster closely with their respective global reference strains, confirming the recent reclassification of the former *Staphylococcus sciuri* group into *Mammaliicoccus* and validating its applicability across geographically diverse isolates.

The *tuf* gene‐based phylogenetic tree revealed two well‐supported clades corresponding to *M. lentus* and *S. simulans*. The Sudanese isolates clustered closely with their respective global reference strains, demonstrating high genetic similarity within each species. This clustering pattern supports the recent reclassification of the former *S. sciuri* group into the genus *Mammaliicoccus* and confirms the consistency of this taxonomic revision among geographically diverse isolates (Figure 7).

## 4 Discussion

This study was conducted to identify the CNS and *Mammaliicoccus* species isolated from caprine milk using conventional and molecular biology methods. These species are present as normal flora on the skin surface and mucous membranes of humans and animals (Piette and Verschraegen 2009). *S. simulans and M. lentus* species were isolated from raw caprine milk. The presence of *S. simulans* and *M. lentus* in raw caprine milk is of particular interest. *S. simulans* is an opportunistic pathogen associated with IMIs and, as identified in this study, may harbor *mecA*, which contributes to methicillin resistance. *M. lentus*, formerly *S. lentus*, is commonly found in small ruminants and carries resistance determinants or biofilm‐forming abilities, thus being of concern for persistence within the udder and transferring resistance genes. These findings therefore emphasize the regular monitoring of CNS in dairy herds and apply the prudent use of antimicrobials to limit the spread of resistance through the food chain.

This study found that identifying CNS using conventional bacteriological methods effectively differentiated species based on biochemical characteristics. The flowchart designed by El Sanousi et al. (2015) was followed. Isolates were identified as *Staphylococcus simulans* and *Mammaliicoccus lentus*, formerly classified under the genus *Staphylococcus*. Despite its reclassification, *M. lentus* maintained identical phenotypic features to those previously associated with *S. lentus*, supporting the notion that the taxonomic shift is not reflected at the phenotypic level (Goering 2023). This finding reinforces the utility of classical diagnostic tools in routine identification, especially in settings with limited access to molecular methods.

Flow Chart for Identification of *Staphylococcus* spp. Developed by El Sanousi and others (El Sanousi et al. 2015), this guide was utilized in conventional bacteriology tests to identify CNS in the present study. Identification of the isolates following this flow chart was 100% identical to that obtained by PCR and partial sequencing of the *tuf* gene. However, for a more comprehensive evaluation, the use of reference strains for all staphylococci is needed to assess their accuracy, as the number of species identified here is small.

Phenotypically, both species exhibited traits that align with prior reports in the literature. *M. lentus* was novobiocin‐resistant, oxidase‐ and urease‐negative, and capable of fermenting raffinose. *S. simulans*, by contrast, displayed novobiocin sensitivity and similar negative reactions for oxidase and urease. These differences in novobiocin susceptibility, though subtle, provide reliable markers for species‐level differentiation (Becker et al. 2014). The absence of phylogenetic divergence observed between *S. lentus* and *M. lentus* underlines the fact that taxonomy is dynamic, and reclassification may not necessarily correlate with significant phenotypic or ecological variation.

Methicillin susceptibility testing revealed that all isolates were susceptible to methicillin, except for *S. simulans*, which exhibited phenotypic resistance. This finding is clinically relevant, as CNS have been increasingly recognized as reservoirs of antimicrobial resistance genes, particularly *mecA*, which confers resistance to β‐lactam antibiotics (Otto 2013). The detection of methicillin resistance in *S. simulans* is consistent with previous studies that reported variable resistance patterns among CNS isolated from raw milk and dairy products (Perales‐Adán et al. 2018; Výrostková et al. 2021). This highlights the importance of continuous surveillance, particularly for foodborne isolates that may serve as reservoirs of resistance genes capable of horizontal transfer to more pathogenic species.

*M. lentus* (previously *S. lentus*) has been isolated in large populations from domestic sheep and goats (Kloos et al. 1976) and occasionally from other domestic animals. It is considered a zoonotic pathogen with the potential to form biofilm, which complicates the recovery of patients in hospitals (Al‐Azawi et al. 2018). This isolate was 99.6% closer to the *M. lentus* strain Sle‐091lar (accession no. MG557993.1) from Greece. One *S. simulans* was identified in this study from caprine milk; it was phenotypically resistant to methicillin, and the methicillin resistance gene (*mecA* gene) was confirmed by PCR. *The tuf* gene sequence of *S. simulans* isolated in this study was 99.6% similar to that of the reference strain *S. simulans* strain PCM 2106 (accession no. MF679022.1) and *S. simulans* strain CJ16 (accession no. KP325407.1); both strains from China.

The phylogenetic analysis based on the Maximum Likelihood method revealed that the *Staphylococcus simulans* isolate from Sudan clusters within a clade comprising *S. simulans* strains IVB and DRD, indicating a close evolutionary relationship. However, the Sudanese isolate forms a distinct branch, suggesting minor genetic divergence and possible regional variation. This clade is separate from another well‐supported cluster that includes strains PCM 2106, N, and CJ16. Within the analyzed group, the reference strain NCTC11046 appears to be the most distantly related (Becker et al. 2014; Pizauro et al. 2021). The overall topology of the tree highlights the genetic diversity among *S. simulans* strains and supports the unique placement of the Sudanese isolate within the species.

The radial phylogenetic tree constructed for *Mammaliicoccus lentus* reveals distinct genetic lineages among the analyzed strains. The Sudanese isolate forms an independent branch, indicating notable divergence from other strains, particularly PCM2441 and strain 7048. While some strains like SJe0911ar exhibit minimal divergence, the Sudanese strain demonstrates a clear evolutionary separation, supporting the existence of regional variation or unique genomic features. Overall, the tree highlights the phylogenetic diversity within *M. lentus*, underscoring the novelty and potential epidemiological importance of the Sudanese isolate.

These molecular results confirmed the preliminary identification obtained via conventional microbiological methods, thereby validating the phenotypic and biochemical identification approaches used in this study. The accurate identification through partial *tuf* gene sequencing reinforces the reliability of combining both phenotypic and genotypic methods for staphylococcal and Mammaliicoccal classification (Becker et al. 2014).

A phylogenetic tree constructed using the elongation factor Tu (*tuf*) gene was developed to investigate the genetic divergence between the genera *Staphylococcus* and *Mammaliicoccus*, specifically focusing on isolates obtained from Sudan in comparison with global reference strains from GenBank. This combined analysis was conducted to assess how the recent taxonomic classification, *Staphylococcus sciuri* group, which is now defunct, has been split into the newly defined genus *Mammaliicoccus*. This reclassification applies to local Sudanese isolates, and their genetic profiles support it (Madhaiyan et al. 2020). The resulting tree reveals two distinct clades: one representing *Staphylococcus simulans*, with the Sudanese isolate clustering closely with global *S. simulans* strains, and the other representing *Mammaliicoccus lentus*, where the Sudanese isolate groups tightly with reference strains such as PCM2441 and Sle091lar. The clear genetic separation between these clades supports the evolutionary divergence of the two genera and validates the accuracy of current classifications (Heikens et al. 2005). This analysis not only confirms the taxonomic placement of the Sudanese isolates but also provides molecular evidence that the revised classification is applicable and consistent across geographically diverse isolates (Cavanagh et al. 2012). Isolation of *S. simulans* and *M. lentus* from raw caprine milk is of importance, considering that both species are emerging CNS involved in subclinical mastitis and could serve as a reservoir for antimicrobial resistance genes. This highlights the microbial diversity of caprine milk and its possible One Health implications. The phylogenetic footprint reflects genetic relatedness and evolutionary pathways between global strains. The clustering of *S. simulans* shows regional adaptation, whereas the strong genomic conservation of *M. lentus* indicates genomic stability among geographical locations. This represents a further contribution to epidemiological surveillance and justifies further studies on the evolution of antimicrobial resistance in dairy microbiota.

## 5 Conclusion and Recommendations

This study is the first in Sudan to identify CNS species from bovine and caprine milk using both conventional bacteriological methods and molecular techniques. The identification approach described by El Sanousi and his colleagues (El Sanousi et al. 2015) was found to be effective and reliable for distinguishing staphylococcal species with high accuracy.

The findings underscore the importance of using phenotypic and molecular techniques in the accurate diagnosis of bacteria and in profiling their antimicrobial resistance. While conventional methods are accessible and practical, the emergence of methicillin‐resistant CNS, such as the *S. simulans* strain identified in this study, highlights the need for molecular diagnostics to monitor antimicrobial resistance in the food chain. However, the study did not investigate the toxins or virulence factors of the isolated CNS species. Further research is recommended to determine their potential roles in animal infections, particularly mastitis, and their possible contribution to foodborne diseases. Moreover, molecular typing of the detected *mecA* gene is advised to trace the origin of methicillin resistance and assess its public health implications.

## Author Contributions

**Ahmed B. Omer:** conceptualization (lead), writing – original draft (equal), formal analysis (equal), writing – review and editing (equal). **Kamal H. Eltom:** conceptualization (lead), writing – original draft (supporting), writing – review and editing (supporting). **Adam Bashir Tawor:** conceptualization (supporting), formal analysis (lead), writing and editing (lead). **Osman Erganiş:** conceptualization (supporting), formal analysis (supporting), writing and editing (supporting).

## Ethics Statement

This study was approved by the SVC, animal care and use committee (approval no: SVC012001908). All procedures complied with national/institutional regulations and adhered to the principles of the 3Rs.

## Conflicts of Interest

The authors declare no conflicts of interest.

## References

[mbo370171-bib-0001] Al‐Azawi, I. H. , A. H. Al‐Hamadani , and S. O. Hasson . 2018. “Association Between Biofilm Formation and Susceptibility to Antibiotics in *Staphylococcus lentus* Isolated From Urinary Catheterized Patients.” Nano Biomedicine and Engineering 10, no. 2: 97–103. 10.5101/nbe.v10i2.p97-103.

[mbo370171-bib-0002] Becker, K. , C. Heilmann , and G. Peters . 2014. “Coagulase‐Negative Staphylococci.” Clinical Microbiology Reviews 27, no. 4: 870–926. 10.1128/cmr.00109-13. 25278577 PMC4187637

[mbo370171-bib-0003] Belhout, C. , R. Elgroud , and P. Butaye . 2022. “Methicillin‐Resistant *Staphylococcus aureus* (MRSA) and Other Methicillin‐Resistant Staphylococci and *Mammaliicoccus* (MRNaS) Associated With Animals and Food Products in Arab Countries: A Review.” Veterinary Sciences 9, no. 7: 317. 10.3390/vetsci9070317. 35878334 PMC9320237

[mbo370171-bib-0004] Bernier Gosselin, V. , S. Dufour , P. R. F. Adkins , and J. R. Middleton . 2019. “Persistence of Coagulase‐Negative Staphylococcal Intramammary Infections in Dairy Goats.” Journal of Dairy Research 86, no. 2: 211–216. 10.1017/S0022029919000311. 31138341

[mbo370171-bib-0005] Carter, G. P. , J. Ussher , A Goncalves Da Silva , et al. 2018. “Genomic Analysis of Multiresistant *Staphylococcus capitis* Associated With Neonatal Sepsis.” Antimicrobial Agents and Chemotherapy 62, no. 11: e00898‐18. 10.1128/aac.00898-18. 30150477 PMC6201123

[mbo370171-bib-0006] Cavanagh, J. P. , C. Klingenberg , A. M. Hanssen , et al. 2012. “Core Genome Conservation of *Staphylococcus haemolyticus* Limits Sequence Based Population Structure Analysis.” Journal of Microbiological Methods 89, no. 3: 159–166. 10.1016/j.mimet.2012.03.014. 22484086

[mbo370171-bib-0007] Claeys, W. L. , S. Cardoen , G. Daube , et al. 2013. “Raw or Heated Cow Milk Consumption: Review of Risks and Benefits.” Food Control 31, no. 1: 251–262. 10.1016/j.foodcont.2012.09.035.

[mbo370171-bib-0008] Clark, S. , and M. B. Mora García . 2017. “A 100‐year Review: Advances in Goat Milk Research.” Journal of Dairy Science 100, no. 12: 10026–10044. 10.3168/jds.2017-13287. 29153153

[mbo370171-bib-0032] El Sanousi, S. M. , K. B. Said , S. G. Elbager , A. Awad , K. Rodwan , and K. Eltom . 2015. “A Flow Chart for the Identification of *Staphylococcus* Species.” Journal of Veterinary Medicine and Animal Production 6, no. 2: 93–97.

[mbo370171-bib-0009] Gebremedhin, E. Z. , A. B. Ararso , B. M. Borana , et al. 2022. “Isolation and Identification of *Staphylococcus aureus* From Milk and Milk Products, Associated Factors for Contamination, and Their Antibiogram in Holeta, Central Ethiopia.” Veterinary Medicine International 2022, no. 1: 6544705. 10.1155/2022/6544705. 35574151 PMC9106507

[mbo370171-bib-0010] Gelasakis, A. I. , A. S. Angelidis , R. Giannakou , G. Filioussis , M. S. Kalamaki , and G. Arsenos . 2016. “Bacterial Subclinical Mastitis and Its Effect on Milk Yield in Low‐Input Dairy Goat Herds.” Journal of Dairy Science 99, no. 5: 3698–3708. 10.3168/jds.2015-10694. 26898280

[mbo370171-bib-0011] Getahun, Y. A. , S. L. Abey , A. M. Beyene , M. A. Belete , and T. S. Tessema . 2024. “Coagulase‐Negative Staphylococci From Bovine Milk: Antibiogram Profiles and Virulent Gene Detection.” BMC Microbiology 24, no. 1: 263. 10.1186/s12866-024-03415-0. 39026151 PMC11256419

[mbo370171-bib-0012] Goering, R. , H. M. Dockrell , M. Zuckerman , and P. L. Chiodini . 2023. Mims' Medical Microbiology E‐Book: Mims' Medical Microbiology E‐Book. Elsevier Health Sciences.

[mbo370171-bib-0013] Goetz, C. , Y. D. N. Tremblay , D. Lamarche , et al. 2017. “Coagulase‐Negative Staphylococci Species Affect Biofilm Formation of Other Coagulase‐Negative and Coagulase‐Positive Staphylococci.” Journal of Dairy Science 100, no. 8: 6454–6464. 10.3168/jds.2017-12629. 28624271

[mbo370171-bib-0014] Heikens, E. , A. Fleer , A. Paauw , A. Florijn , and A. C. Fluit . 2005. “Comparison of Genotypic and Phenotypic Methods for Species‐Level Identification of Clinical Isolates of Coagulase‐Negative Staphylococci.” Journal of Clinical Microbiology 43, no. 5: 2286–2290. 10.1128/jcm.43.5.2286-2290.2005. 15872257 PMC1153770

[mbo370171-bib-0015] Kloos, W. E. , K. H. Schleifer , and R. F. Smith . 1976. “Characterization of *Staphylococcus sciuri* sp. nov. and Its Subspecies.” International Journal of Systematic and Evolutionary Microbiology 26, no. 1: 22–37. 10.1099/00207713-26-1-22.

[mbo370171-bib-0016] Koop, G. , S. De Vliegher , A. De Visscher , et al. 2012. “Differences Between Coagulase‐Negative *Staphylococcus* Species in Persistence and in Effect on Somatic Cell Count and Milk Yield in Dairy Goats.” Journal of Dairy Science 95, no. 9: 5075–5084. 10.3168/jds.2012-5615. 22916911

[mbo370171-bib-0017] Koop, G. , T. van Werven , H. J. Schuiling , and M. Nielen . 2010. “The Effect of Subclinical Mastitis on Milk Yield in Dairy Goats.” Journal of Dairy Science 93, no. 12: 5809–5817. 10.3168/jds.2010-3544. 21094753

[mbo370171-bib-0039] Kumar, S. , G. Stecher , M. Suleski , M. Sanderford , S. Sharma , and K. Tamura . 2024. “MEGA12: Molecular Evolutionary Genetic Analysis Version 12 for Adaptive and Green Computing.” Molecular Biology and Evolution 41, no. 12: msae263. 10.1093/molbev/msae26. 39708372 PMC11683415

[mbo370171-bib-0018] Kumar, S. , G. Stecher , M. Suleski , M. Sanderford , S. Sharma , and K. Tamura . 2024. “MEGA12: Molecular Evolutionary Genetic Analysis Version 12 for Adaptive and Green Computing.” Molecular Biology and Evolution 41, no. 12: msae263. 10.1093/molbev/msae263. 39708372 PMC11683415

[mbo370171-bib-0019] Madhaiyan, M. , J. S. Wirth , and V. S. Saravanan . 2020. “Phylogenomic Analyses of the Staphylococcaceae Family Suggest the Reclassification of Five Species Within the Genus *Staphylococcus* as Heterotypic Synonyms, the Promotion of Five Subspecies to Novel Species, the Taxonomic Reassignment of Five *Staphylococcus* Species to *Mammaliicoccus* Gen. Nov., and the Formal Assignment of *Nosocomiicoccus* to the Family Staphylococcaceae.” International Journal of Systematic and Evolutionary Microbiology 70, no. 11: 5926–5936. 10.1099/ijsem.0.004498. 33052802

[mbo370171-bib-0020] Martineau, F. , J. Picard , P. H. Roy , M. Ouellette , and M. G. Bergeron . 1998. “Species‐Specific and Ubiquitous‐DNA‐Based Assays for Rapid Identification of *Staphylococcus aureus* .” Journal of Clinical Microbiology 36, no. 3: 618–623. 10.1128/jcm.36.3.618-623.1998. 9508283 PMC104596

[mbo370171-bib-0021] Mohamedsalih, S. A. , and E. A. A. Abubaker . 2021. “Bacterial Quality of Goat Raw Milk in Khartoum State, Sudan.” Asian Journal of Research in Animal and Veterinary Sciences 8, no. 4: 106–111.

[mbo370171-bib-0022] Nei, M. , and S. Kumar . 2000. Molecular Evolution and Phylogenetics. Oxford University Press.

[mbo370171-bib-0023] Nelli, A. , C. C. Voidarou , B. Venardou , et al. 2022. “Antimicrobial and Methicillin Resistance Pattern of Potential Mastitis‐Inducing *Staphylococcus aureus* and Coagulase‐Negative Staphylococci Isolates From the Mammary Secretion of Dairy Goats.” Biology 11, no. 11: 1591. 10.3390/biology11111591. 36358292 PMC9687969

[mbo370171-bib-0024] Nemeghaire, S. , W. Vanderhaeghen , M. A. Argudín , F. Haesebrouck , and P. Butaye . 2014. “Characterization of Methicillin‐Resistant *Staphylococcus* Sciuri Isolates From Industrially Raised Pigs, Cattle, and Broiler Chickens.” Journal of Antimicrobial Chemotherapy 69, no. 11: 2928–2934. 10.1093/jac/dku268. 25063778

[mbo370171-bib-0025] Ogodo, A. C. , D. I. Agwaranze , M. Daji , R. E. Aso , et al. 2022. “Microbial Techniques and Methods: Basic Techniques and Microscopy.” In Analytical Techniques in Biosciences, 201–220. Elsevier. 10.1016/B978-0-12-822654-4.00003-8.

[mbo370171-bib-0026] Otto, M. 2013. “Coagulase‐Negative Staphylococci as Reservoirs of Genes Facilitating MRSA Infection.” BioEssays: News and Reviews in Molecular, Cellular and Developmental Biology 35, no. 1: 4–11. 10.1002/bies.201200112. 23165978 PMC3755491

[mbo370171-bib-0027] Perales‐Adán, J. , S. Rubiño , M. Martínez‐Bueno , et al. 2018. “LAB Bacteriocins Controlling the Food Isolated (Drug‐Resistant) Staphylococci.” Frontiers in Microbiology 9: 1143. 10.3389/fmicb.2018.01143. 29946300 PMC6005826

[mbo370171-bib-0028] Pickering, A. C. , G. Yebra , X. Gong , et al. 2021. “Evolutionary and Functional Analysis of Coagulase Positivity Among the Staphylococci.” mSphere 6, no. 4. 10.1128/msphere.00381-21. PMC8386474 34346700

[mbo370171-bib-0029] Piette, A. , and G. Verschraegen . 2009. “Role of Coagulase‐Negative Staphylococci in Human Disease.” Veterinary Microbiology 134, no. 1–2: 45–54. 10.1016/j.vetmic.2008.09.009. 18986783

[mbo370171-bib-0030] Pizauro, L. J. L. , C. C. de Almeida , S. R. Silva , et al. 2021. “Genomic Comparisons and Phylogenetic Analysis of Mastitis‐Related Staphylococci With a Focus on Adhesion, Biofilm, and Related Regulatory Genes.” Scientific Reports 11, no. 1: 17392. 10.1038/s41598-021-96842-2. 34462461 PMC8405628

[mbo370171-bib-0031] Saitou, N. , and M. Nei . 1987. “The Neighbor‐Joining Method: A New Method for Reconstructing Phylogenetic Trees.” Molecular Biology and Evolution 4, no. 4: 406–425. 3447015 10.1093/oxfordjournals.molbev.a040454

[mbo370171-bib-0033] Schauer, B. , M. P. Szostak , R. Ehricht , et al. 2021. “Diversity of Methicillin‐Resistant Coagulase‐Negative *Staphylococcus* spp. and Methicillin‐Resistant *Mammaliicoccus* spp. Isolated From Ruminants and New World Camelids.” Veterinary Microbiology 254: 109005. 10.1016/j.vetmic.2021.109005. 33582485

[mbo370171-bib-0034] Strommenger, B. , C. Kettlitz , T. Weniger , D. Harmsen , A. W. Friedrich , and W. Witte . 2006. “Assignment of *Staphylococcus* Isolates to Groups by Spa Typing, Smai Macrorestriction Analysis, and Multilocus Sequence Typing.” Journal of Clinical Microbiology 44, no. 7: 2533–2540. 10.1128/jcm.00420-06. 16825376 PMC1489514

[mbo370171-bib-0035] Suresh, R. 2020. Histopathological and Immunohistochemical Studies on Bovine Calf Diarrhoea. Guru Angad Dev Veterinary and Animal Sciences University.

[mbo370171-bib-0036] Tamura, K. , and M. Nei . 1993. “Estimation of the Number of Nucleotide Substitutions in the Control Region of Mitochondrial DNA in Humans and Chimpanzees.” Molecular Biology and Evolution 10, no. 3: 512–526. 10.1093/oxfordjournals.molbev.a040023. 8336541

[mbo370171-bib-0037] Virdis, S. , C. Scarano , F. Cossu , V. Spanu , C. Spanu , and E. P. L. De Santis . 2010. “Antibiotic Resistance in *Staphylococcus aureus* and Coagulase‐Negative Staphylococci Isolated From Goats With Subclinical Mastitis.” Veterinary Medicine International 2010, no. 1–6: 517060. 10.4061/2010/517060. 20445785 PMC2860459

[mbo370171-bib-0038] Výrostková, J. , I. Regecová , F. Zigo , B. Semjon , and G. Gregová . 2021. “Antimicrobial Resistance of *Staphylococcus* sp. Isolated From Cheeses.” Animals: An Open Access Journal from MDPI 12, no. 1: 36. 10.3390/ani12010036. 35011142 PMC8749609

